# Augmented reality and optical navigation assisted orbital surgery: a novel integrated workflow

**DOI:** 10.1515/iss-2023-0064

**Published:** 2024-07-29

**Authors:** Nikolay Tonchev, Giulia Renieri, Klaus-Peter Stein, Belal Neyazi, Max Willgerodt, Hagen Thieme, I. Erol Sandalcioglu, Karl Hartmann

**Affiliations:** Universitätsklinik für Neurochirurgie, Otto-von-Guericke-Universität Magdeburg, Leipziger Str. 44, 39120 Magdeburg, Germany; Universitätsklinik für Augenheilkunde, Otto-von-Guericke-Universität Magdeburg, Leipziger Str. 44, 39120 Magdeburg, Germany

**Keywords:** orbital surgery, intraoperative optical navigation, augmented reality, orbital cavernous malformation, minimally invasive microsurgery

## Abstract

**Objectives:**

Due to the close topographical relationship of functional relevant anatomic structures, limited space and cosmetic aspects orbital surgery will remain a challenging discipline. Therefore, novel technical capabilities are necessary for further surgical progress. We here tested the integration of augmented reality and optical navigation in one workflow for interdisciplinary decision-making, feasibility and intraoperative guidance.

**Methods:**

High-resolution contrast-enhanced MRI and CT scans were automated and manual-assisted segmented to achieve a detailed three-dimensional (3D) model of the individual patho-anatomical relationships. Augmented reality was used for interdisciplinary preoperative planning and intraoperative intuitive navigation. Mayfield clamp head holder in combination with optical surface matching registration assured navigation assisted microsurgery.

**Results:**

Combinations of different MRI-sequences and CT-scans were necessary for detailed 3D-modeling. Modeling was time consuming and only viable in the hands of medical, surgical and anatomical trained staff. Augmented reality assured a quick, intuitive interdisciplinary orientation. Intraoperative surface matching registration enabled precise navigation in the orbital space.

**Conclusions:**

Optical Navigation and microscope integration achieved a straightforward microsurgical workflow and should be implemented regularly. Augmented reality represented a useful tool for preoperative interdisciplinary planning and intraoperative intuitive orientation. It further stated an excellent educational tool.

## Introduction

Orbital tumors are rare pathologies in close relation to delicate and functional relevant anatomic structures. Detailed preoperative considerations should be an interdisciplinary assignment with radiological, ophthalmological and neurosurgical expertise to set the required approach and optimize the surgical workflow [1], [2], [3], [4], [5]. In the past, implementation of novel technical abilities leads to an improvement of the surgical workflow and procedural morbidity [6], [7], [8], [9], [10], [11]. Therefore, novel techniques should also be tested in the domain of orbital surgery. We here focused on (A) three-dimensional augmented reality (AR) visualization of individual patho-anatomic relations for interdisciplinary decision-making, (B) novel tools for intraoperative guidance to delineate concealed delicate structures and (C) integration of the surgical microscope in image-guided surgical workflows.

For 3D visualization, augmented reality (AR) could state a unique tool. So far, AR states the only visual technique, which allows for a holographic representation of individual medical scans in a 3D space with realistic dimensions [12]. For surgical personnel in the demanding field of orbital surgery this could be of outstanding value for intuitive preoperative anticipation of crucial surgical steps.

The basis for such detailed simulations of individual anatomy and pathological alterations in the AR space are precise 3D models [13], 14]. These models need to be based on adequate imaging sets. We here suggested combining multimodal imaging to pay respect to the operative conditions of the orbit.

CT scans could adequately delineate the fine architecture of the orbit and juxta-proximate osseous structures of the frontal and temporal cranial base and facial bones [15]. MRI should adequately delineate the various soft tissues of the orbit [16]. T1 contrast-enhanced imaging should be relevant for vasculature and tumor representation. Heavily T2 weighted imaging sequences like fast imaging employing steady-state acquisition (FIESTA) or constructive interference in steady state (CISS) could well delineate crucial orbital structures like orbital muscles [17], nerves [18] and lacrimal glands [19].

Afterwards, these neuroimaging data sets need to be combined to achieve one coherent data set. Here automated and manually controlled image fusion achieves a precise overlay of different imaging modality data [20], 21]. Only then, a direct comparison of tissue characteristics is possible. The combination of T1 contrast enhanced and heavily T2 weighted imaging sequences could e.g. differentiate fine vasculature from orbital nerves [22].

3D modeling can then be achieved via segmentation of 3D data sets [23], 24]. Here automated and manually controlled as well as solely manual segmentation of anatomical and pathological structures by specialists, who are trained in orbital anatomy and surgery is required. With multiple AR glasses, an interdisciplinary case discussion in AR space is possible.

Optical stereotactic techniques could assure quick and precise image registration in orbital surgeries since the face of the patient is mostly bare and in an optimal position to the stereotactic cameras. Image and microscope registration would then allow for a projection of the 3D model as a hologram in the operative microsurgical field [25], 26].

Recently augmented reality and mixed reality were introduced into orbital surgery within different treatment procedures with promising clinical outlook. A first technical note on implementation of AR for canthal ligament alignment procedures was published in 2011 by Mezzana et al. [27]. An application here measured the preoperative, intraoperative and postoperative lateral canthal angle position by projecting lines above the patients face. In 2021 Liu et al. published a technical description of an AR-Workflow for orbital floor reconstruction procedures, which was successfully integrated and achieved reduced rates of implant malposition and thus decrease the surgical risk for the patient [28]. Similar procedure with step-by-step description of its implementation was described by a case report from Zoabi et al. The authors incorporated AR during the implantation of a patient specific implant in case of an orbital floor fracture to control for implant positioning [29]. Following original publications focused on the use of AR mainly in reconstructive operations. Carpinello et al. examined three different scenarios in maxillofacial surgery in order to provide an evaluation of the VR head-mounted displays and highlight the lacking features such as optical zoom, blood flow live visualization and others [30]. Zhu et al. described in his publication another successful application of AR in treatment of orbital hypertelorism [31]. Furthermore, it is stated that AR technology could represent a helpful tool for precise osteotomy in craniofacial surgery. A recent systematic review from Dubron et al. presented the current amount of scientific experience and came to the conclusion that virtual reality-based educational tools provide better visualization and understanding of craniofacial trauma compared to conventional 2- or 3-dimentional images [32]. Generally, all these publications come to the conclusion that AR could find many different medical implications, in controlling optical accuracy of implant positioning and facial proportions after reconstructive operations. Extended clinical data sets, proving this seem to be missing so far. The scope of this article focuses on the translation of AR technology in neoplastic orbital surgery for preoperative interdisciplinary case discussion, intraoperative intuitive navigation with lenses and integration into the operative microscope during tumor resection.

## Materials and methods

### Medical history

A 65-years-old female patient was diagnosed with a right intraorbital lesion 7 years ago. The patient initially had personal restraints according to surgical management. At the time of consultation in our orbital center she presented with progressive exophthalmus, double vision in the last two years, intermittent orbital pain and vertigo. Cardiac arrhythmia and depression were co-existing diseases. The further patient history was free of malignancies or relevant diagnoses.

The inspection revealed exophthalmus and downward displacement of the right eyeball with decent ciliary injection, see Figure 4. An abnormal head position was present with the chin rotated upwards.

The ophthalmological examination proved good/best corrected visual acuity (20/25 ft or 0.1 LogMAR) on both eyes with normal visual fields (Humphrey Field Analyzer 3, Carl Zeiss Meditec, SITA Standard 24-2 with a white size III stimulus on a white background). Double vision was noted in upwards and left gaze with restriction of adduction and elevation of the right eye due to compression of *M. rectus* medialis, *M. rectus* superior and *M. obliquus* superior. Lang test showed normal results. An anterior eye segment exam revealed a mild sclerotic cataract in both eyes, while the posterior eye segment inspection was unremarkable.

**Figure 1:**
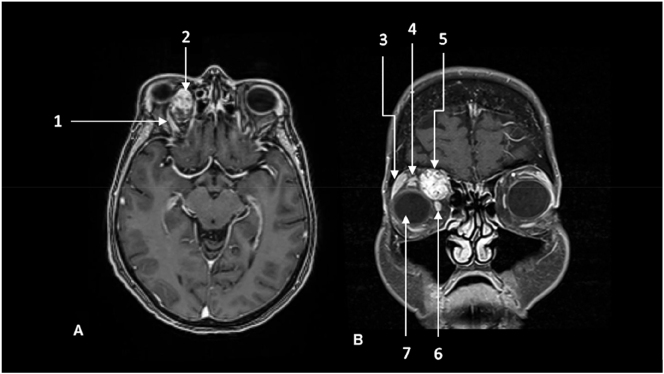
Preoperative MRI scans depicting the tumor of the right orbit. (A) axial T1-weighted sequence, 1 mm, 3D, contrast enhanced. Note the intraconal oval hyperintense tumor of the right nasal orbit with compression of the eyeball, optic nerve, orbital muscles and consecutive exophthalmos. (1) *M. rectus* superior, (2) tumor, (B) coronal T1-weighted sequence, 1 mm, 3D, contrast enhanced, with adequate delineation of the tumor. Further, note the edematous and compressed *M. rectus* medialis and *M. rectus* superior in contrast to the unaffected orbital muscles on the left. Further, note that *M. obliquus* superior is elevated and compressed superiorly and flattened against the periorbit and orbital roof. Note the dilated lacrimal gland on the right side in comparison to the left side as an indirect sign of a compression of the upper lid excretory ducts. (3) Lacrimal gland, (4) *M. rectus* superior, (5) tumor, (6) *M. rectus* medialis, (7) globe.

The patient underwent cranial CT- and MRI-imaging. This showed a retrobulbar intraconal globe-shaped lesion with a diameter of 27 x 20 mm and a relocation of the Bulbus oculi, *N. opticus*, *M. rectus* medialis, *M. rectus* superior, *M. obliquus* superior and orbital vasculature, see Figure 1 and Video I (see supplementary material). We interpreted the lesion as a progressive orbital cavernous malformation and recommended surgical resection.

### Preanalytic imaging

For clear depiction of orbit and juxta proximate osseous structures high-resolution CT with 0.75 mm slice-thickness was performed and restricted to the orbit and facial bones. This allowed reduction of radiation dose (KVP: 100 kV, 41/50 mA s/ref., DLP: 46 mGy cm, Ti: 1.0 s, cSL: 0.6 mm; Slice thickness 0.75 mm; Pixel size 0.35 × 0.35 mm^2^).

For delineation of vessel and tumor volume cranial MRI with and without gadolinium contrast was executed. Native T1 sequences with a slice thickness of 1.00 mm³ isotropic voxel size were used for registration of intraoperative navigation and trajectory planning as well as contrast enhanced T1 sequences for tumor and lacrimal gland segmentation, volume extraction and vascular analysis. Additionally, CISS-sequences enabled further anatomical localization of ocular muscles, optic nerve and lacrimal gland; see Table 1 for detailed sequence characteristics.

**Table 1:** MRI characteristics.

	MRI sequence	FA (flip angle, deg)	TR (repetition time, ms)	TE (echo time, ms)	TI (inversion time, ms)	Slice thickness (ST, mm)
MRI head ± contrast	T2 TSE axial	150°	4,850.00	97.00		5.00 mm
	T1 MPRAGE post-C sagittal with coronal and axial reconstruction	8°	1,570.00	2.65	900.00	1.00 mm

MRI orbit ± contrast	T2 TSE coronal	150°	3,190.00	84.00		3.00 mm
	T1 TSE coronal	90°	259.00	8.80		3.00 mm
	T2 CISS 3D axial with sagittal reconstruction	62°	5.36	2.41		0.70 mm
	T1 TSE FS post-C coronal	160°	518.00	10.00		3.00 mm

Siemens Aera^®^ 1.5 T: MRI head ± contrast agent: T2 turbo-spin-echo axial, T1 MPRAGE post-contrast sagittal with coronal and axial reconstruction. MRI orbit ± contrast agent: T2 turbo-spin-echo coronal, T1 turbo-spin-echo coronal, T2 CISS 3D axial with sagittal reconstruction, T1 turbo-spin-echo fat-suppressed post-contrast coronal.

### 3D modeling

First, CT-scans, MRI T1 native whole head 1 mm 3D, MRI T1 contrast enhanced whole head and MRI heavily T2 weighted CISS orbital sequences were auto-fused and manually corrected. Second, distortion correction of MRI scans with the CT 1 mm as a template was performed. Third, crucial anatomical structures were automated segmented in the specific modality. Skin reconstruction with T1, 1 mm, 3D; skull, orbit and juxta proximate cranial base with CT and T1, 1 mm, 3D; eyeball, lens, orbital muscles and N. II, optic nerve with heavily weighted T2 CISS sequences; tumor matrix, orbital and tumorous vessels and lacrimal apparatus with T1 1 mm, 3D contrast enhanced; branches of optic motor nerve; trochelar nerve, abducens nerve with heavily weighted T2 CISS and T1 contrast enhanced sequences.

Here it is to be noted that automated segmentation failed if imaging showed artifacts, was blurred or anatomic structures were not well delineated in the primary sequence. Automated segmentation further failed in the juxta proximity of the tumor and in aberrations of anatomy due to tumor dislocation or compression. Though automated segmentation was extensively time saving it needed meticulous control and adaptation with manual segmentation in the hands of trained staff in cranial imaging and surgery. Finally, it is to be noted that templates for anatomic structures of the orbit are still rare, which of course inhibits automated segmentation.

**Figure 2:**
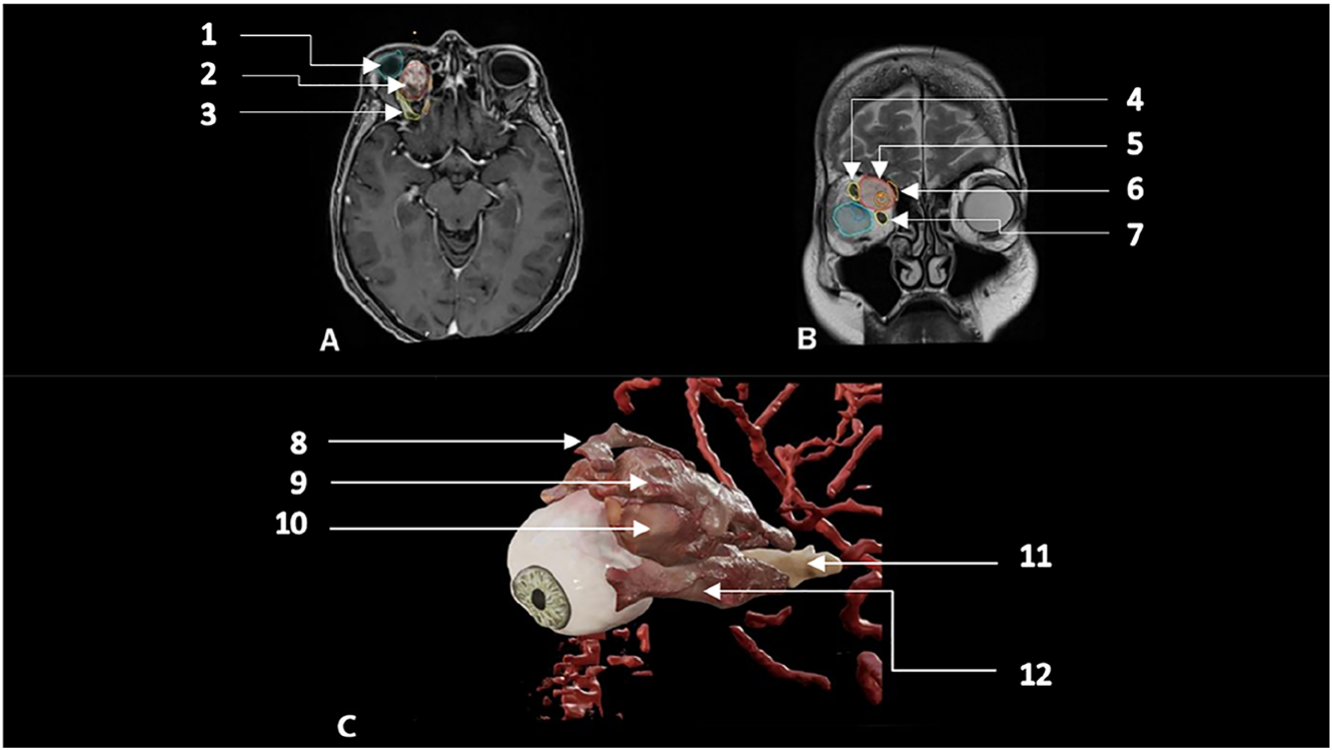
Preoperative 3D-modeling. Relevant surgical structures are displayed. (A) Axial- and (B) coronal-T1-weighted, contrast enhanced sequence with automated and manual segmented relevant surgical structures. (1) Globe, (2) tumor mass, (3) *M. rectus* superior, (4) *M. rectus* superior, (5) tumor, (6) *M. obliquus* superior, (7) *M. rectus* medialis, (C) 3D volumetric model of segmented structure on the level of orbit. (8) *M. obliquus* superior, (9) *M. rectus* superior, (10) tumor, (11) optic nerve and (12) *M. rectus* medialis.

In the end of this processing we achieve a 3D volume model of the tumor and relevant proximate structures, see Figure 2 and Video I (see supplementary material).

The operative trajectory was planned after an interdisciplinary case discussion and plotted in the 3D model. Here, an approach inferior to the trochlear of *M. obliquus* superior, medial to the *M. rectus* superior and superior to the *M. rectus* medialis was chosen.

The different steps were performed with BrainLab software (BrainLab Elements™: Image Fusion, Cranial Segmentation, Distortion Correction, Smart Brush, Image Viewer and Trajectory Planning).

### Preoperative augmented reality

In order to achieve a realistic simulation of the conditions during the operation, a skull model was used to adjust the size and patient positioning of the holographic 3D model. The hologram was manually adapted to the skull model by using characteristic osseous structures like nasion and bilateral mastoid tips as well as the overall surface of the skull (see Video I, supplementary material). In this manner the interdisciplinary team could achieve an intuitive 3D representation of the positional relationships of crucial anatomic and pathologic structures to enhance case discussion. Augmented reality (AR) was achieved with Magic Leap 1 glasses (Lumin OS, 1,280 × 960 pixel RGB per eye, ROM 128 GB, RAM 8 GB) and Brainlab^®^ Mixed Reality Viewer.

### Intraoperative augmented reality

During the operation, the skull model was placed in the OR and the hologram was adapted as described. After patient positioning and head fixation the hologram was manually adjusted to the skin of the face and characteristic osseous structures like nasion and mastoid tips. This enabled an overlay of the 3D model with the operative trajectory directly over the patient’s head for intuitive orientation and interdisciplinary planning of each step of the operation, see Video I (supplementary material).

### Intraoperative frameless stereotactic navigation

The head of the patient was placed in the Mayfield head holder. The frameless stereotactic navigation was established. Surface matching registration was used with registration points at the facial ridges and edges close to the orbit, at the orbital rim and on the medial sagittal and coronal line of the skull. Registration was verified. The surgical microscope was registered to the navigational system, allowing an overlay of the 3D model in the view of the surgeon and displaying the point of the microscope’s focus in the neuroimaging data set. Navigation was performed with “Curve Navigation System, Brainlab” and microscopy with “Kinevo, Zeiss Co”, see Video I (supplementary material).

### Surgical steps

**Figure 3:**
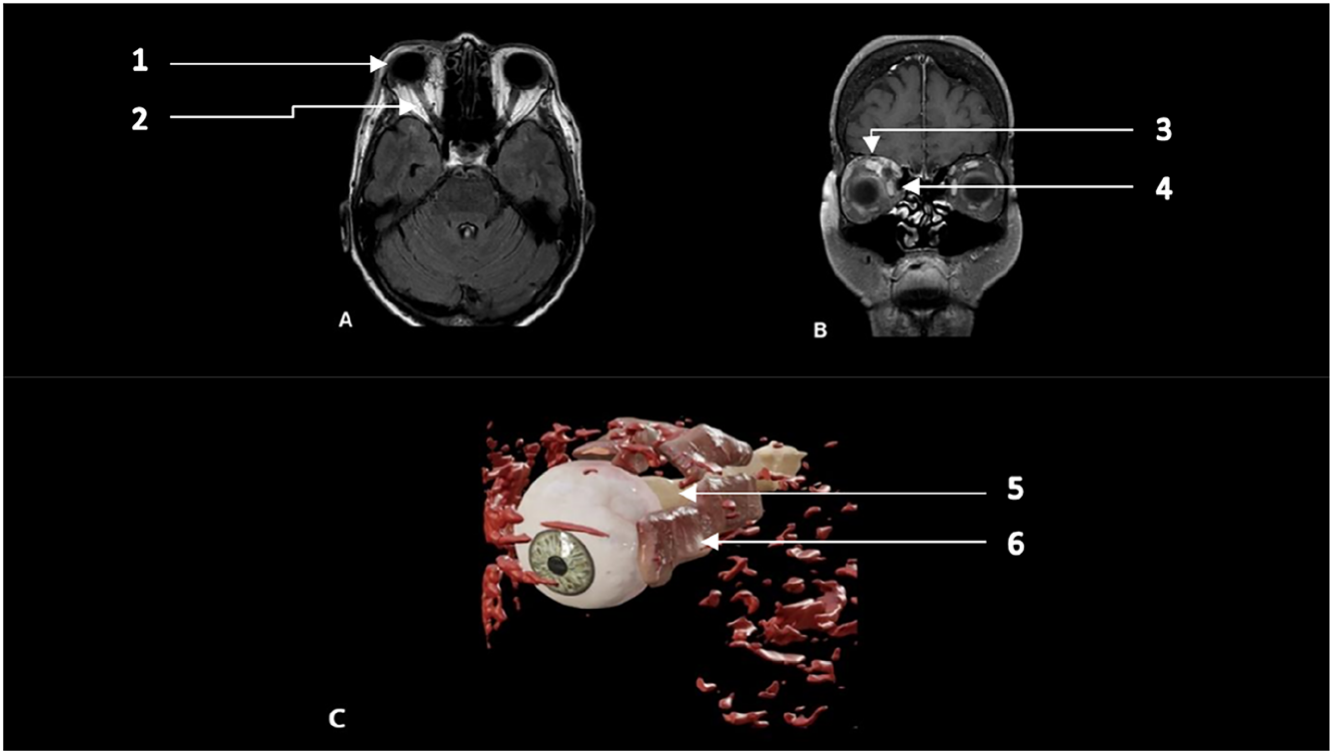
Postoperative MRI and Volume Reconstruction. (A) Axial and (B) coronal T1-weighted image with contrast on the level of orbit. (1) Globe, (2) optic nerve, (3) *M. rectus* superior, (4) *M. rectus* medialis. (C) Volume reconstruction of eyeball and orbital muscles. (5) optic nerve, (6) *M. rectus* medialis.

**Figure 4:**
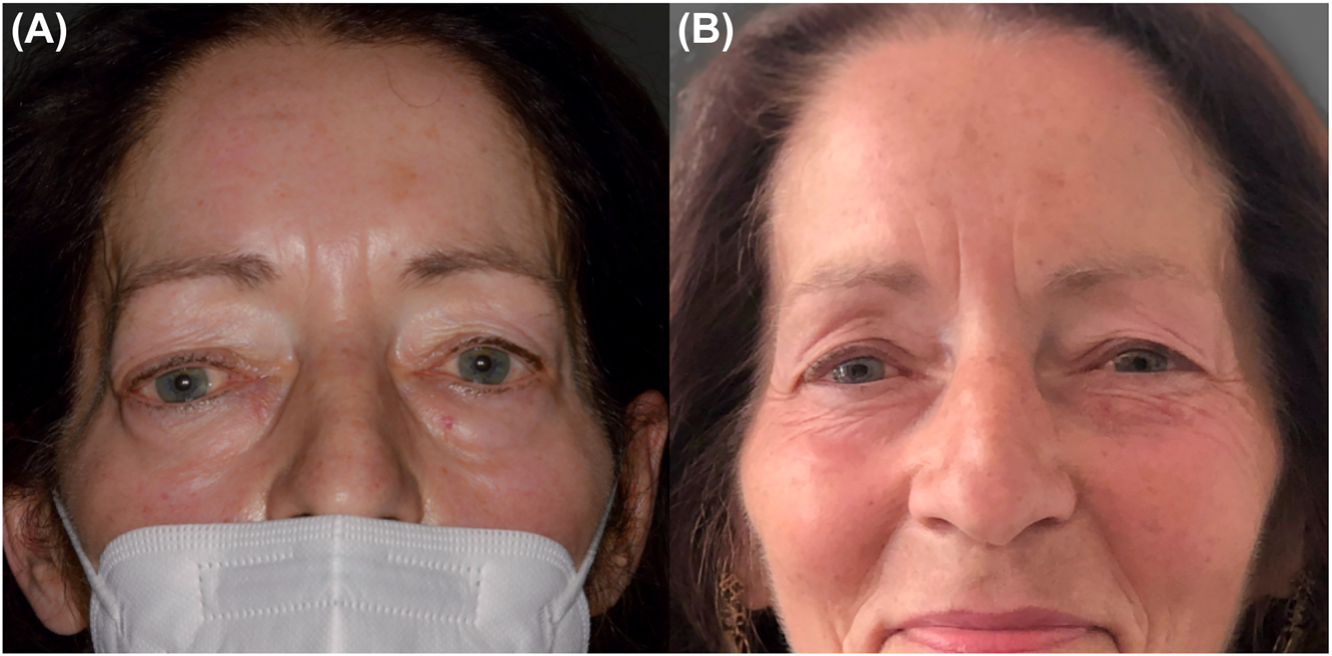
Pre- and postoperative medical examination. (A) Preoperative portrait photography demonstrating a right basal bulbus deviation, upper and lower lid edema and exophthalmus. (B) Postoperative portrait photography demonstrating a recovered bulbus position, lid edema and exophthalmus. Note the minimal invasive upper lid incision with an excellent cosmetic result.

After head positioning, a 1 cm linear incision was undertaken in the medial third of the upper eyelid. The intraoperative microscope-assisted navigation proved the exact localization and showed the planned trajectory. Further preparation and blunt dissection of the periorbital fat was performed, without the necessity of performing a craniotomy. Leyla retractor with ribbon brain spatula helped to sustain desired surgical corridor in order to reach the intraconal lesion. After exposure of a circumscribed area of the mass, consecutive coagulation under constant irrigation and smooth mobilization allowed shrinkage of the lesion. The main supplying blood vessels of the tumor were branches of the ethmoidal and ophthalmic arteries. These were identified and dissected after careful coagulation. The tumor could then be removed in toto. The tumor cavity between m. obliquus superior and m. rectus superior was then inspected for subtle bleeding. Wound closure was performed with subcutaneous sutures and Steri-Strip adhesives. Postoperative MRI proved complete tumor removal and decompression of periorbital structures see Figure 3 and 4 and Video I (see supplementary material).

Immediately postoperative double vision and exophthalmus was not present anymore. No novel neurological or visual deficits have emerged. On the 2nd postoperative day, moderate eye lid edema occurred. The ophthalmologic examination proved preserved direct and consensual pupillary reflex to light and stable visual acuity. The histologic examination confirmed the diagnosis of cavernous malformation of the orbit. The patient was discharged home on the 5th postoperative day.

## Results

The above-described preoperative scanning protocol assured an adequate delineation of relevant anatomy and pathology. Here it is to be noted that whole head 3D 1 mm T1 MRI scans are necessary for skull and skin representation to align the hologram to the skull model and patients face with surface matching. Contrast enhancement was relevant for tumor delineation and differentiation of vessels and orbital nerves such as e.g. the supratrochlear artery and the trochlear nerve. Heavily T2 weighted 1 mm CISS sequences were relevant for detailed depiction of fine orbital anatomy and could be limited to the orbit to save scanning time. This protocol enabled straightforward fusion of image data, which was necessary for the following creation of the 3D model. Furthermore, the limitations of the neuroimaging capabilities have to be taken into account. Although exact localization of both vascular (ophthalmic artery, ethmoidal artery) and neural structures (optic nerve and proximal aspects of the oculomotor nerve) was possible, the delineation of intraconal segments of the oculomotor nerve, trochlear nerve and abducens nerve was difficult. We suppose that MRI-imaging capabilities in regard to distal nerve branches, penetrating the intraocular muscle fascias, will remain limited.

3D-modeling occurred to be time consuming since manual control of automated segmentation and detailed manual segmentation in the hand of trained professionals was necessary. Approximately 8 h were needed for detailed neuro-anatomical depiction. The reasons for this are first, that automated segmentation algorithms to determine the fine anatomic structures of the orbit are simply missing. Second, our analysis proved that the existing automated segmentation algorithms needed manual correction and verification, done by trained specialist in the domain of neuroanatomy, orbital surgery and neuroimaging. And third, the automated segmentation algorithms tend to fail in the case of mass effects, complex individual anatomy and pathologies, because the algorithms were primarily trained with average, non-pathological datasets. However, by training and repetition as well as future automated segmentation methods for orbital structures, procedure time could probably be reduced [33].

Augmented reality 3D-model inspection is an intuitive and unique tool for case discussion and interdisciplinary preoperative planning. It further supports the education of medical students and residents [34]. Finally, it states an excellent tool to realize the spatial relationship of the lesion and functional relevant anatomic structures.

During the phase of patient positioning and head fixation, AR glasses could be used for interdisciplinary intuitive intraoperative orientation and case discussion, if the hologram was correctly aligned with the patient’s skull. Here 3D skin modeling was useful for alignment with facial characteristics and 3D skull modeling with alignment of classic anatomical osseous orientation points like the nasion and mastoid tips.

During the phase of minimal invasive surgery, intraconal orientation will remain challenging because of narrow working corridors, functionally relevant delicate structures and pathological alterations of individual anatomy [3], 4]. Here intraoperative optical navigation helped to delineate priorly concealed anatomic structures. One of the main obstacles for precise optical navigation is correct registration. In the case of orbit, surface-matching registration can be easily applied. Accuracy will be high due to the multiple angles and ridges of the juxta-proximate facial surface [35]. Apart from the orbital region, it was mandatory to use a medial sagittal and coronal line of registration points to enhance precision in the depth of the operative corridor.

3D-modeling in combination with surface matching registration led to AR applications via the microscope during the microsurgical workflow [14]. In real time, 3D-models could be blended over the surgical site in the view of the surgeon. Furthermore, the position of the microscope focus into the neuroimaging data set could be defined via head-up displays.

In orbital cavernous malformation surgery, tumor shrinkage is a key step. However, it consecutively leads to tissue shift, which restrained our image guidance at this point. Intraoperative ultrasound could be used for re-registration here.

## Conclusions

According to our experience AR has shown to be beneficial for preoperative interdisciplinary case discussion. Here the spatial relationship between the pathology and the functional relevant anatomic structures can be visualized in 3D and viewed from different angles. Thus, the surgical team can discuss the approach and the crucial steps of the operation in a simulated environment. AR here states a tool for intuitive comprehension of operative anatomy. By means of simulation, young students could faster achieve the necessary awareness of the local anatomy in highly complex surgical cases such as meningiomas of the skull base and intracavernous lesions. Therefore, we emphasize that this technique should be used regularly as an educational tool for students, residents and medical specialists in various domains.

The illustrated clinical case was used to test this novel integrated workflow into real medical practice. On one hand, AR enabled anticipation of the complex anatomy of the orbital cavity. On the other hand, intraoperative frameless optical navigation could be effortlessly integrated through surface-matching registration within the facial area. Using the combination of both technologies could therefore be very useful also in more complex pathologies e.g. cranio-maxillofacial surgery [31] and facial plastic surgery.

Intraoperative AR still has the restriction of manual registration. Here technical improvements to AR glasses should make use of the integrated stereo-laser surface scanning, which would enable automated surface matching registration. Similar technology already exists [36] and its modification could lead to an automated alignment of the holographic 3D model and the patient’s skin surface. It could further improve registration precision, which is the base for valid navigation. The lacking possibility of real object magnification, such as that of microscope technique, has to be seen as additional restriction of AR in the use in orbital cavity. Nevertheless, by the combination of lighter and less cost-intensive AR glasses, usability would rise and greater medical expertise could be gained.

## Supplementary Material

Supplementary Material
