# Supplementary material for: Augmented reality and optical navigation assisted orbital surgery: a novel integrated workflow
Source: Innov Surg Sci. 2024 Jul 29;10(2):91–8. doi: 10.1515/iss-2023-0064 (PMC12327852; doi:10.1515/iss-2023-0064)
Supplement: Supplementary file 1 — Supplementary Material [file j_iss-2023-0064_suppl_001.docx]

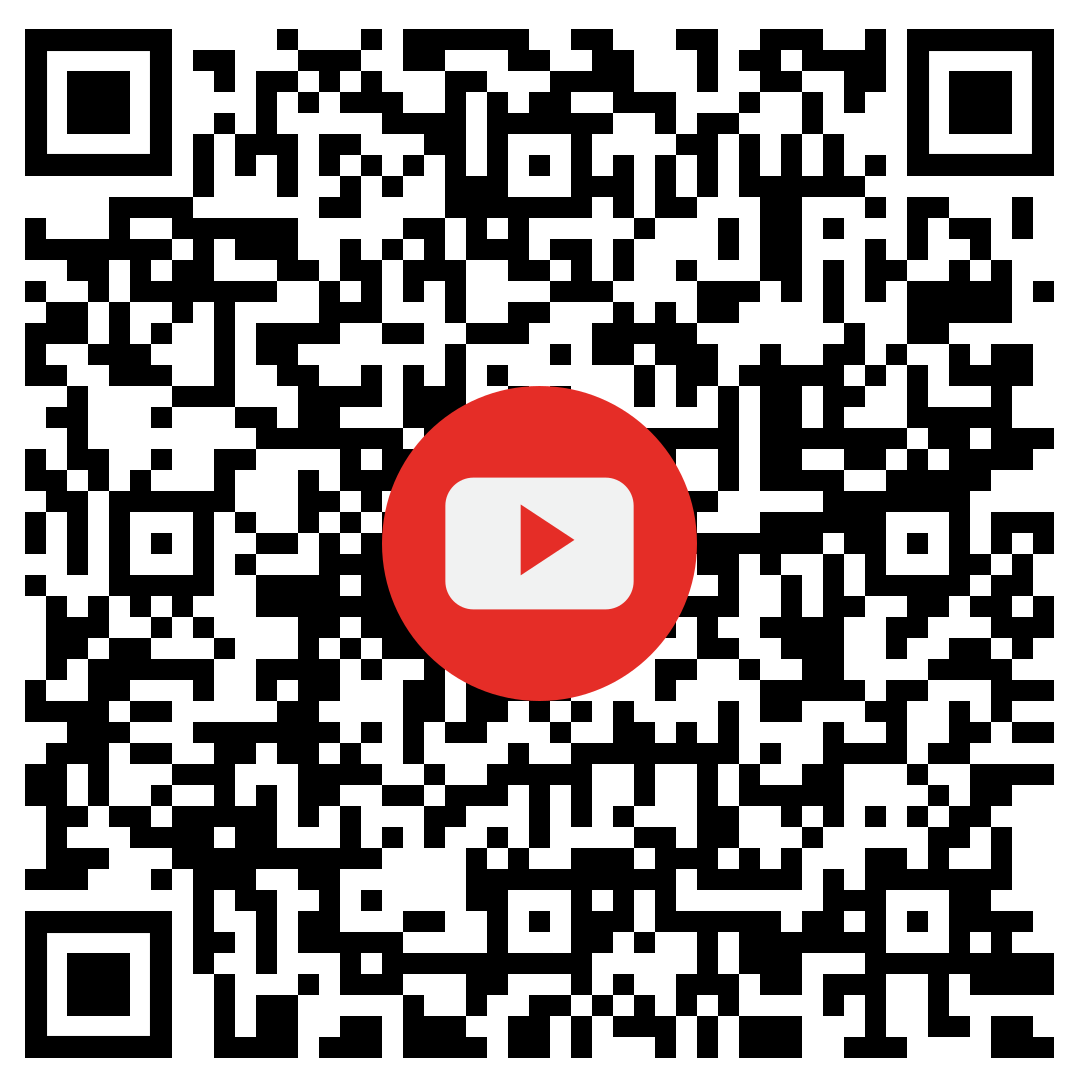
**<-- scan to play** [**Video I**](https://drive.google.com/file/d/1XX5emhTjQtga8H_ObyDmozvw3P9t9LTV/view?usp=sharing)

**Video I** Augmented Reality and Navigation Assisted Orbital Surgery. Pre- and postoperative augmented reality inspection of 3D model and intraoperative surgical workflow demonstrating integration of augmented reality and navigation in the microsurgical workflow to achieve a minimally invasive resection of a slow flow orbital cavernous malformation.
